# Network-Based Assessment of Adverse Drug Reaction Risk in Polypharmacy Using High-Throughput Screening Data

**DOI:** 10.3390/ijms20020386

**Published:** 2019-01-17

**Authors:** Guillermo de Anda-Jáuregui, Kai Guo, Junguk Hur

**Affiliations:** 1 Computational Genomics Division, National Institute of Genomic Medicine (INMEGEN), Alcaldía de Tlalpan, Ciudad de México C.P. 14610, Mexico; gdeanda@inmegen.edu.mx; 2 Department of Biomedical Sciences, University of North Dakota School of Medicine and Health Sciences, Grand Forks, ND 58202, USA; kai.guo@med.und.edu

**Keywords:** network pharmacology, adverse drug reaction, polypharmacology, polypharmacy, risk prediction, Library of Integrated Network-Based Cellular Signatures, LINCS, L1000 assay

## Abstract

The risk of adverse drug reactions increases in a polypharmacology setting. High-throughput drug screening with transcriptomics applied to human cells has shown that drugs have effects on several molecular pathways, and these affected pathways may be predictive proxy for adverse drug reactions. Depending on the way that different drugs may contribute to adverse drug reactions, different options may exist in the clinical setting. Here, we formulate a network framework to integrate the relationships between drugs, biological functions, and adverse drug reactions based on the high-throughput drug perturbation data from the Library of Integrated Network-Based Cellular Signatures (LINCS) project. We present network-based parameters that indicate whether a given reaction may be related to the effect of a single drug or to the combination of several drugs, as well as the relative risk of adverse drug reaction manifestation given a certain drug combination.

## 1. Introduction

For a drug to be successful, it needs to strike a balance between its therapeutic and toxic effects [1]. Adverse drug reactions (ADRs), broadly defined as harmful or unpleasant reactions resulting from therapeutic interventions, may have negative health and economic consequences [2]. The risk of ADRs increases in the context of polypharmacy, the simultaneous use of multiple different drugs by the same patient in order to treat one or more medical conditions. Polypharmacy is especially common in the elderly population, putting them at higher risk of developing ADRs [3]. Given a therapeutic scheme consisting of several drugs, it is commonly difficult to know how to (1) remove or substitute a drug, and (2) identify the drug that caused the ADR in the first place.

Drug-induced gene expression high-throughput screening (GE-HTS) has generated large datasets containing profiles of the effects of drugs on gene expression in different cellular systems. These datasets can be used to identify the effects of drugs on biological processes involving sets of functionally related genes, such as those annotated in databases of controlled vocabularies such as Gene Ontology (GO) [4] and/or cell signaling, metabolic, and gene regulatory pathway databases. One of the largest publicly available GE-HTS efforts is that of the original Connectivity Map (CMap) [5] and its continuation as part of the Library of Integrated Network-Based Cellular Signals (LINCS) [6,7]. Importantly, through such methods, it is possible to identify multiple targets on which a drug acts (which is known as polypharmacology [8], as opposed to the previously defined polypharmacy). 

Recently, LINCS drug perturbation profiles were used to identify predictive relationships between GO term perturbation and ADRs [9]. These predictive relationships may be represented as a network of associations between functional perturbations (observed as the statistically significant change in overall expression of genes involved in the biological function) and the emergence of ADRs, referred to as the GO–ADR network.

Network analyses from a topological perspective (that is, considering the structures that arise from the way that the elements in the network are connected) are useful in analyzing complex systems. Particularly, in the context of drugs, we have found that multiple-layer network formalisms, where different types of elements belong to different layers and may interact across layers [10], are particularly suited to studying and analyzing pharmacological systems, as these are usually composed of elements of different nature such as drugs and side effects [1].

**Figure 1:**
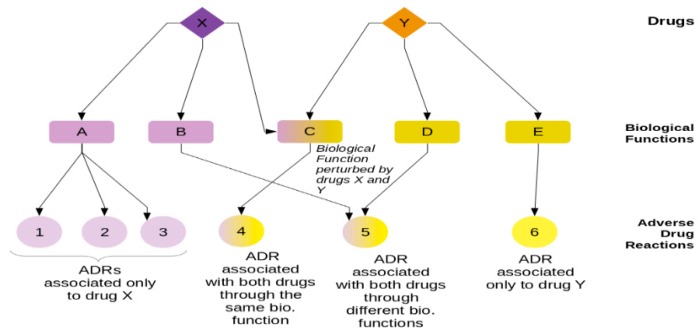
A diagram of three-layer (tripartite) network of drugs, biological functions, and adverse drug reactions (ADRs). This diagram includes two drugs (X and Y), where each drug may perturb biological functions. Drug X perturbs biological functions A and B, while drug Y perturbs biological functions D and E. Both drugs can also perturb function C. Each biological function perturbation may be associated with certain ADRs. Some ADRs are associated only with one drug (ADRs 1, 2, and 3 are only associated with drug X through biological function A, while ADR 6 is associated only with drug Y through biological function E). Some ADRs may be associated with both drugs. ADR 4 is associated with a function that may be perturbed by both drugs, an example of our “mode 1” model in this study. ADR 5 is associated with both drugs through different biological functions B and D, each of which is perturbed by drugs X and Y, respectively. ADR 5 is an example of our “mode 2” model in this study.

In any given set of drugs, each drug may affect different biological functions, either by design or through off-target effects. These perturbations of biological functions may in turn be associated with the manifestation of ADRs, as illustrated in Figure 1. In this clinical case example, two drugs, X and Y, are prescribed. ADRs may be manifested from this combination, where ADRs may only be associated with one of the two drugs. However, there may be some ADRs due to the effects of either or both drugs, making the management of these ADRs more complicated for the physician. The complexity of ADR manifestation increases when more drugs are used simultaneously, which may be better understood through the use of complex network strategies. 

In this work, we have expanded the aforementioned GO–ADR network to include GO perturbation by drugs, modeled as a tripartite network that identifies all paths connecting drugs to ADRs through functional perturbations. We explored the topological features of this network, which may contribute to the understanding of the functional perturbations behind ADRs in a polypharmacy setting. This network provides a useful means to assess whether a given ADR is the result of a single drug or may involve the added or combined effects of multiple drugs. This information may inform clinical decisions regarding treatment management in a polypharmacy setting.

## 2. Results

### 2.1. Topological Properties of the Tripartite Network

**Figure 2:**
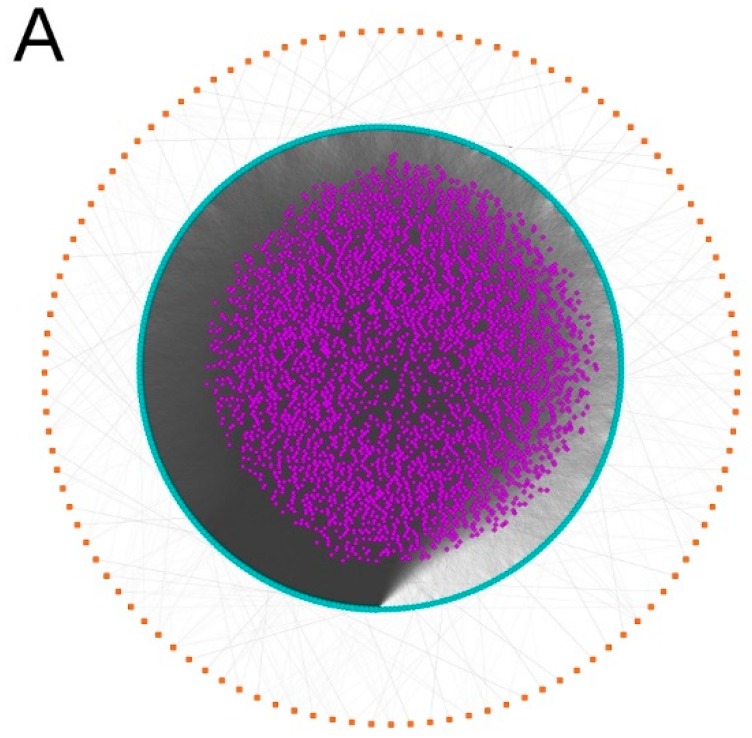

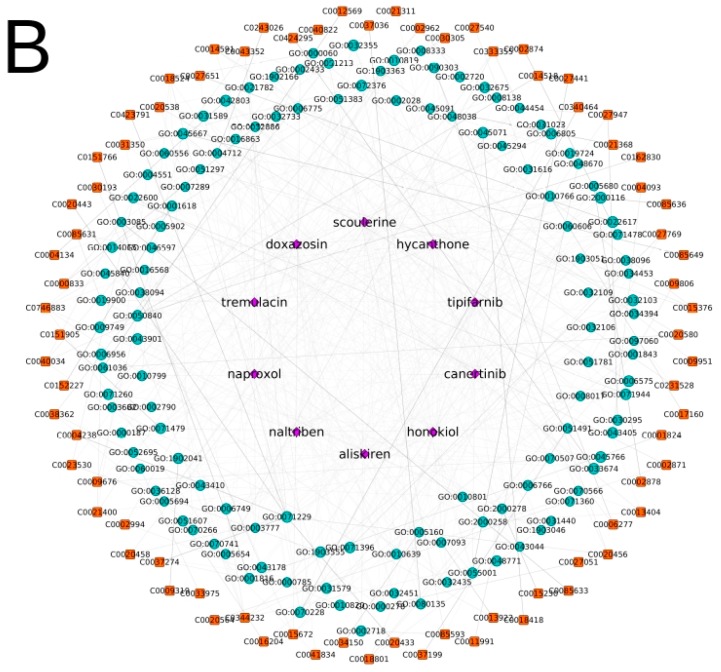
Tripartite Drug–GO–ADR network. Drugs are represented by purple diamonds, which are connected to Gene Ontology (GO) terms, represented by blue circles, which may be perturbed by the drug. In turn, GO terms are connected to those adverse drug reactions (ADRs), represented by red squares, that may manifest if said GO term is perturbed. In panel (**A**), the full network is shown. Panel (**B**) shows a subgraph of 10 (arbitrarily selected) drugs with their associated GO terms and ADRs.

We obtained the published LINCS-based GO–ADR network generated by Wang et al. [9] and expanded it by adding drug nodes and connecting these nodes to the GO terms that they perturb (see Methods for details). The resulting network is a tripartite directed network with three types of nodes: DRUG nodes, GO nodes, and ADR nodes. It is also a directed acyclic graph (DAG) that consists of paths of length 2 (DRUG→GO→ADR); each path represents the possible perturbation of a GO term by a drug, and the possible emergence of the ADR given the GO term perturbation. Figure 2A illustrates the complete network, whose topological properties are summarized in Table 1, and a partial network of 10 arbitrarily selected drugs is visualized in Figure 2B. The complete network file is available as Supplementary File 1 in GML format.

### 2.2. Topological Properties of Drug Combination Subgraphs

Given a drug combination consisting of two or more drugs, a subgraph may be obtained of the tripartite network presenting the landscape of perturbable GO terms and ADRs associated with that drug combination. In this study, we defined four concepts that may be useful to explore these drug combination subgraphs: composite ADRs (cADR; ADRs that may be associated with more than one drug in the combination), configuration mode 1 (ADRs associated with pathways that may be perturbed by more than one drug in the combination), configuration mode 2 (ADRs associated with pathways perturbed by different drugs in the combination), and composite risk modules (CRMs; sets of pathways and ADRs that may be functionally related to each other). The use of a network formalism allows the identification of connectivity patterns that may be systematically evaluated to inform about different risks associated with a different combination.

**Figure 3:**
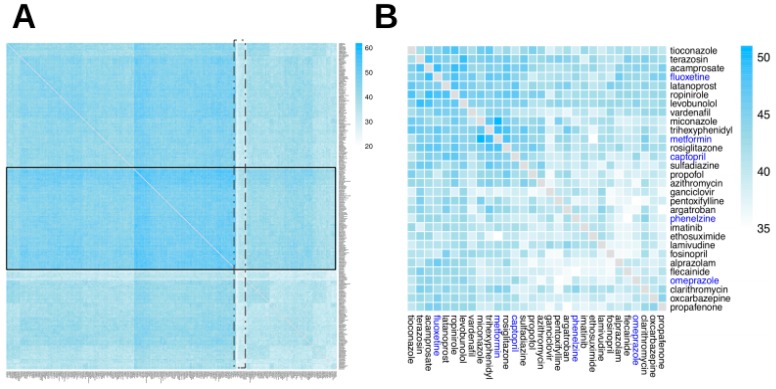
Composite ADR heatmap. Heatmap of two-drug combination composite ADRs (cADRs). Composite ADRs are reactions that may be caused by any of the drugs in the combination. The heatmap is organized using a hierarchical clustering (a connectivity-based grouping method, in which a set of dissimilarities is generated from the original matrix data, and each drug is iteratively assigned to a cluster). (**A**) The complete heatmap, containing all 315 drugs; a band of drugs with more cADRs (highlighted in a solid line rectangle), as well as a narrow band of drugs with few cADRs (highlighted in a dotted rectangle) are shown. (**B**) A subset of the heatmap, containing 35 drugs: five drugs highlighted in blue, which are used in examples in the Discussion section, and the six drugs most similar to each of them (in terms of the number of cADRs) from Panel A.

The number of composite ADRs (cADRs) for a given drug combination indicates how many ADRs may be the result of the action of any (or all) of the drugs in the combination. Figure 3 shows a heatmap of cADRs for two-drug combinations (for 315 drugs that were included in the GO–ADR network and annotated in the PharmGKB dataset used in a previous study [11]) and suggests that the existence of cADRs between drugs is common, with only a few drug pairs exhibiting a low number of cADRs and a large cluster of two-drug combinations with high (over 50) numbers of cADRs.

**Figure 4:**
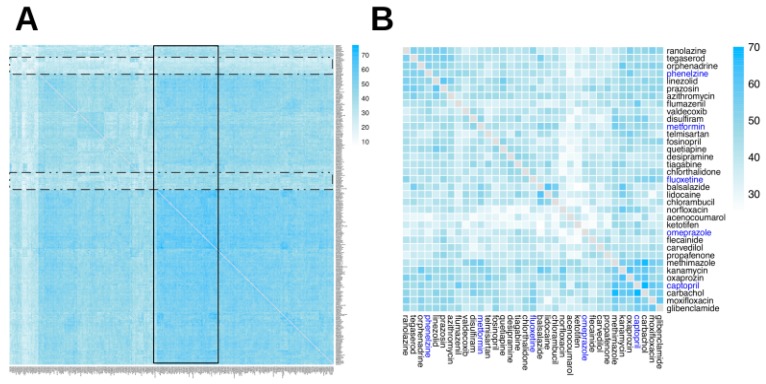
Heatmap of two-drug combination cADRs via mode 1 configuration. The mode 1 configuration indicates that the two drugs in the combination may perturb the same GO term, which in turn may lead to cADR manifestation. The heatmap is organized using a hierarchical clustering. The heatmap indicates that most drug pairs have a large number of mode 1 configurations. (**A**) The complete heatmap, containing all 315 drugs; two narrow bands of drugs with few mode 1 configurations (dotted rectangles) and a wide band of drugs with many mode 1 configurations (solid line rectangle) are shown. (**B**) A subset of the heatmap, containing 35 drugs: five drugs highlighted in blue, which are used in examples in the Discussion section, and the six drugs most similar to each of them (in terms of the number of instances of mode 1 configurations) from Panel A.

**Figure 5:**
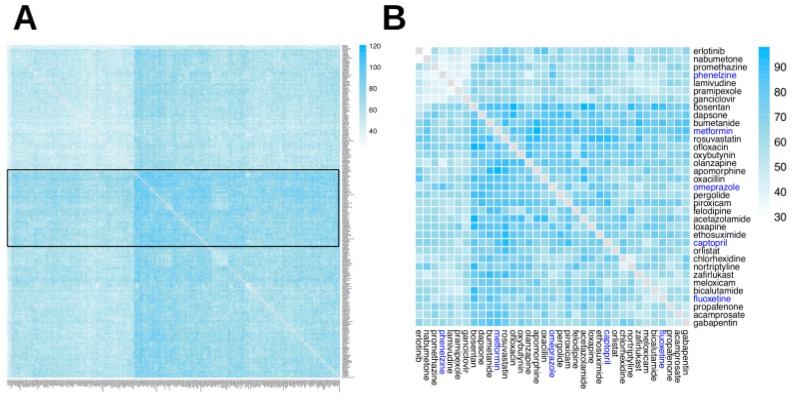
Heatmap of two-drug combination cADRs via mode 2 configuration. The mode 2 configuration indicates that each drug in the combination may perturb a different GO term that is associated with cADR manifestation. The heatmap is organized using a hierarchical clustering. It can be seen that most of most drug pairs exhibit a large number of mode 2 configurations. (**A**) The complete heatmap, containing all 315 drugs; a band of drugs with the most mode 2 configurations is shown (inside the solid line rectangle). (**B**) A subset of the heatmap, containing 35 drugs: five drugs highlighted in blue, which are used in examples in the Discussion section, and the six drugs most similar to each of them (in terms of number of instances of mode 2 configurations) from Panel A.

We defined two different configuration modes that describe the different ways in which drugs may contribute to ADR manifestation: either by targeting the same GO terms (mode 1) or by targeting different pathways (mode 2). Figure 4 and Figure 5 show differences in the distributions of modes 1 and 2 configurations leading to cADRs, where the mode 1 configuration shows a more clustered organization for drug pairs.

**Figure 6:**
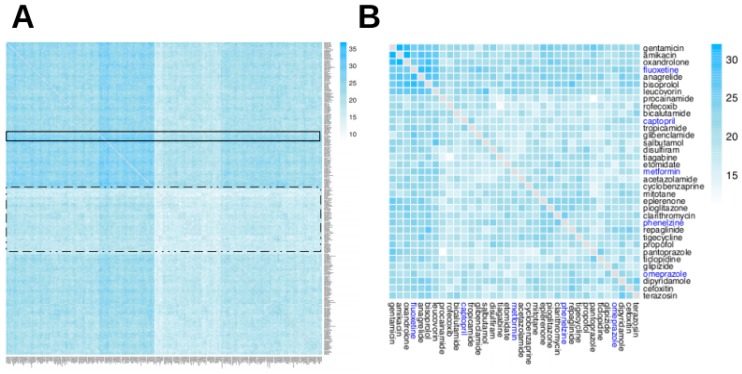
Heatmap of two-drug combination composite risk modules (CRMs). A CRM is a set of GO terms and cADRs that are independently associated in such a way that the manifestation of a cADR in the CRM may only be associated with the perturbation of GO terms in the same CRM. The manifestation of more than one cADR from the same CRM may then be thought to be associated with the perturbation of the same set of GO terms. The heatmap is organized using a hierarchical clustering. (**A**) The complete heatmap, containing all 315 drugs. A narrow band of drugs that exhibit the highest number of CRMs is highlighted (inside a solid line rectangle), as well as a wide band of drugs with a low number of CRMs (inside the dotted line rectangle). (**B**) A subset of the heatmap, containing 35 drugs: five drugs highlighted in blue, which are used in examples in the Discussion section, and the six drugs most similar to each of them (in terms of number of CRMs) from Panel A.

The concept of CRMs may be regarded as a measure of diversity of potentially related ADR sets. Since the ADRs in a given CRM are associated with a shared set of GO terms, it is possible that the manifestation of two or more ADRs from the same CRM may have an origin in the perturbation of a similar GO term (or terms). Figure 6 shows the landscape of such CRMs available for different two-drug combinations.

It must be noted that the heatmaps presented here were generated for two-drug combinations only for simplicity in terms of the illustration of the model. It is possible to use this framework to describe any number of *n*-drug combinations, which will be exemplified in the Discussion section.

The full tripartite network in a graph (GML) format is available as Supplementary File 1, while the analysis codes used in the current study are available in our GitHub repository: https://www.github.com/hurlab/cADR. High-resolution heatmap image files for Figure 3, Figure 4, Figure 5 and Figure 6 are provided as Supplementary File 2.

## 3. Discussion

Our model enables the analysis of drug combinations in terms of the ADRs that may emerge during the course of a drug regime. By considering a wide range of functional perturbations that may be associated with a drug as well as the association of these functional perturbations with the emergence of ADRs, it is possible to systematically categorize the landscape of functional perturbations and ADRs that may be associated with a given drug combination. This was accomplished by modeling them using a tripartite network formalism.

The study of polypharmacy from a systems perspective opens up novel opportunities for clinical applications. A recent work [12] showed that different layers of pharmacological data may be modeled through networks to predict drug interaction side effects. In the present work, we have provided a network formalism that could give quantitative measures of possible interactions of a combination of drugs based exclusively on the data derived from high-throughput drug perturbation experiments. Our work currently does not systematically integrate other sources of drug information, such as chemical structures or known drug targets. Nevertheless, we consider that the formalism here presented provides (1) a possible decision tool for the clinical setting, which may inform physicians of probable mechanisms behind observed adverse drug reactions, and (2) network parameter-based quantitative measures that may form the basis of or complement future comprehensive predictive models. To illustrate these potential applications, we provide the following two examples of drug combinations, analyzed from this network perspective.

### 3.1. Fluoxetine and Phenelzine: Network Analysis of Serotonin Syndrome

**Figure 7:**
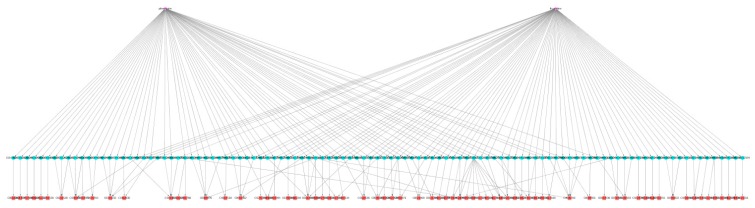
Fluoxetine–phenelzine drug combination network visualization. This is a visualization of the subgraph containing the GO terms and ADRs associated with fluoxetine and phenelzine, two drugs that, in combination, may produce serotonin syndrome. The two drugs are represented by purple diamonds, the 107 GO terms as blue circles, and the 72 ADRs as red squares.

**Figure 8:**
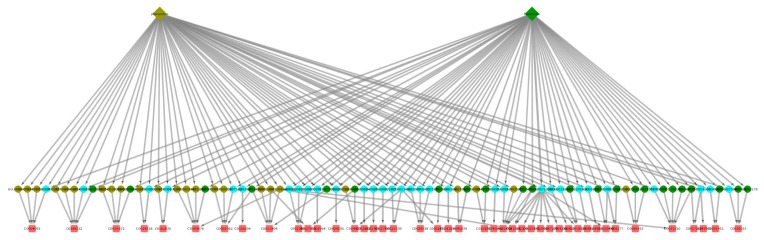
Fluoxetine–phenelzine drug combination network cADR space. This is a visualization of the subgraph containing the cADRs and associated GO terms for the phenelzine and fluoxetine combination. The cADRs are represented as red squares. The two drugs are represented by a light brown and a dark green diamond, respectively. GO terms associated exclusively with phenelzine are represented as light brown circles, and those exclusively associated with fluoxetine as dark green circles, whereas GO terms associated with both drugs are colored blue.

**Figure 9:**
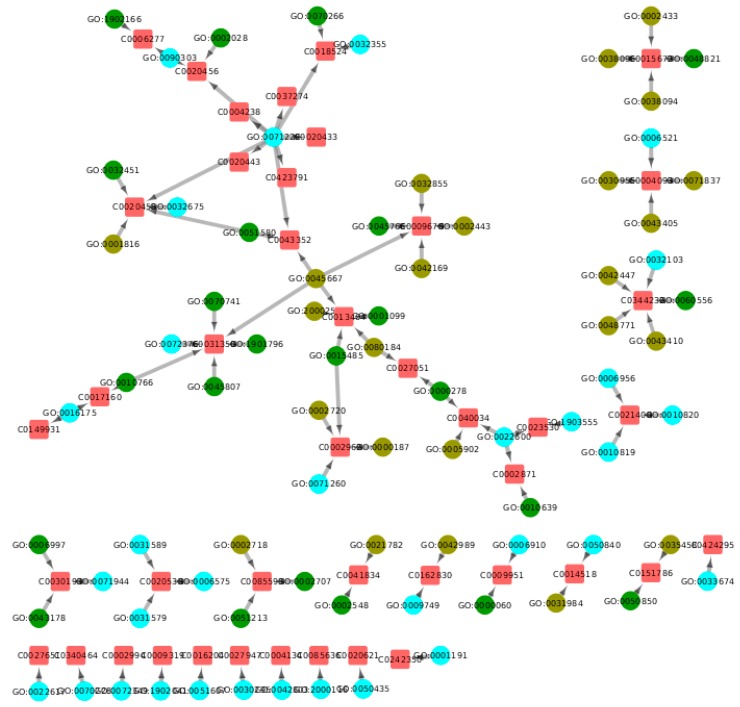
Fluoxetine–phenelzine drug combination network CRMs. This is a visualization of the CRMs for the phenelzine–fluoxetine combination. In this visualization, those GO terms associated only with phenelzine are light brown, those associated only with fluoxetine are dark green, and those associated with both are blue. The red squares represent cADRs. The largest CRM contains 20 different cADRs. The remaining 23 cADRs are independent of each other, each belonging to its own small CRM.

Excessive serotonergic agonism may lead to the appearance of serotonin syndrome, which is a life-threatening condition characterized by the manifestation of several clinical symptoms [13]. In a clinical setting, serotonin syndrome may occur because of the concurrent administration of different serotonergic agonists. We decided to explore the space of ADRs for two such agents—fluoxetine and phenelzine—in order to exemplify our proposed model. Figure 7 shows a subgraph of the Drug–GO–ADR tripartite network focused on the two selected drugs, and Figure 8 shows a subset of the graph including only 43 cADRs and 79 GO terms that are associated with the two drugs. Figure 9 shows the 24 CRMs for this drug combination. Table 2 provides some general parameters for this drug combination.

**Figure 10:**
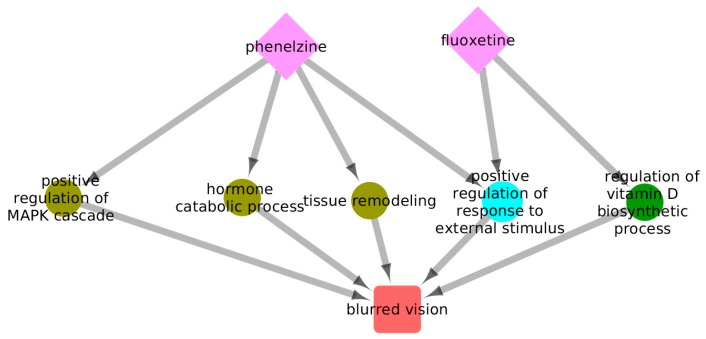
A subgraph of fluoxetine and phenelzine with five pathways. This is an illustration of the manifestation of blurred vision as an ADR to the administration of phenelzine and fluoxetine. Phenelzine and fluoxetine may both perturb the positive regulation of the response to an external stimulus GO term in a mode 1 configuration. Additionally, mode 2 contributions may be observed, with fluoxetine being independently able to perturb the regulation of the vitamin D biosynthetic process and phenelzine being able to perturb the positive regulation of MAPK cascade, hormone catabolic processes, and tissue remodeling. The perturbation of any such GO terms may lead to the manifestation of blurred vision.

Figure 10 illustrates an example of a single ADR, blurred vision, which allows the visualization of contributions to the reaction through modes 1 and 2. In this figure, five GO terms are associated with blurred vision, and how each of these contributes through a different configuration is visualized with arrows.

Three GO terms are potentially perturbed by phenelzine alone, one is potentially perturbed by fluoxetine alone, and only one GO term may be perturbed by both drugs. As we describe in the Methods, it can be demonstrated that, knowing the contribution of each drug to pathway perturbation, as well as the contribution of each individual pathway to the ADR, the activity of this ADR given this two-drug combination can be calculated:

(1) $$\begin{array}{ll} Blurred\;Vision & =Activity\left(BV\;|\;GO:0043410\right)\;\times \;\left[Perturbation\left(GO:0043410\;|\;phenelzine\right)\right]\\ & +\;Activity\left(BV\;|\;GO:0042447\right)\;\times \;\left[Perturbation\left(GO:0042447|\;phenelzine\right)\right]\\ & +\;Activity\left(BV\;|\;GO:0048771\right)\;\times \;\left[Perturbation\left(GO:0048771|\;phenelzine\right)\right]\\ & +\;Activity\left(BV\;|\;GO:0032103\right)\;\times \;[Perturbation\left(GO:0032103|\;phenelzine\right)\\ & +Perturbation\left(GO:0032103\;|\;fluoxetine\right)]\\ & +Activity\left(BV|\;GO:0048771\right)\times \;\left[Perturbation\left(GO:0060556|\;fluoxetine\right)\right] \end{array}$$

### 3.2. Captopril + Metformin + Omeprazole: A Potential Geriatric Combination

**Figure 11:**
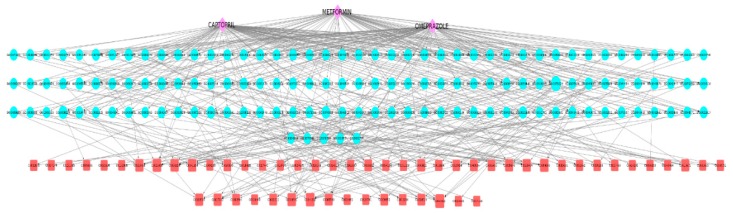
Captopril, metformin, and omeprazole drug combination network visualization. This is a visualization of the subgraph containing the GO terms and ADRs associated with the three drugs. The drugs are represented as purple diamonds, the GO terms as blue circles, and the ADRs as red squares.

**Figure 12:**
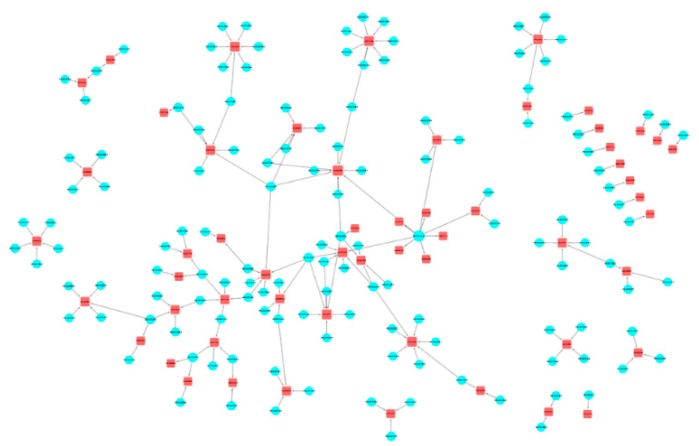
Captopril, metformin, and omeprazole drug combination network CRMs. In this visualization, red squares represent cADRs, and blue circles represent GO terms.

For this final example, we selected three drugs that are widely prescribed in the elderly population. This combination is likely to be frequently used, for instance, by elderly diabetic patients. In Figure 11, we show the space of 56 cADRs and 134 associated GO terms for this drug combination. Figure 12 shows the 21 CRMs associated with this drug combination.

**Figure 13:**
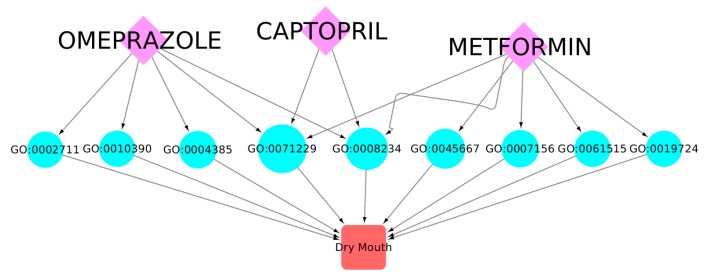
A subgraph of omeprazole, captopril, and metformin and their GO term targets involved in the manifestation of the dry mouth ADR. It may be seen that there are nine different GO terms that, when perturbed by these drugs, may lead to the manifestation of the dry mouth ADR. Three of these GO terms are exclusively perturbed by omeprazole, four exclusively by metformin, and the rest may be perturbed by any of the drugs. Notice there is no GO term that is exclusively associated with captopril.

In Figure 13, we present a subgraph showing the contribution of the three-drug combination to the manifestation of the “dry mouth” ADR [14]. Three GO terms that contribute to dry mouth were associated with omeprazole exclusively: “positive regulation of T-cell-mediated immunity” (GO:0002711), *“*histone monoubiquitination” (GO:0010390), and “guanylate kinase activity” (GO:0004385). Four GO terms were associated exclusively with metformin, including “regulation of osteoblast differentiation” (GO:0045667), “homophilic cell adhesion via plasma membrane adhesion molecules” (GO:0007156), “myeloid cell development” (GO:0061515), and “B cell mediated immunity” (GO:0019724). Interestingly, two GO terms, “cellular response to acid chemical” (GO:0071229) and “cysteine-type peptidase activity” (GO:0008234), were potentially perturbed by any of the three drugs, while there was no dry-mouth-associated GO term that was exclusively perturbed by captopril in this combination.

In this study, we provided a framework to explore the relationships between drugs, functional categories such as GO terms or pathways, and ADRs using a network formalism. We described some topological features that may be evaluated for any drug combination, which may be informative of the way in which these drugs generate adverse drug reactions. This may have both research and clinical applications. For this work, a GO–ADR network was used that was derived from previously published work [9], which identified relationships between drugs, GO terms, and ADRs from HTS perturbation reported in the LINCS dataset; however, our model may be used to explore any set of drugs, functional features, and ADRs available that were derived from other technologies. 

A major strength of our approach is that it may be scaled to analyze any number of drugs; therefore, it is suitable for the analysis of complex therapeutic regimes. Although the visual inspection of such a large network may not be possible at such scale, the computation of the parameters presented in this work is feasible. The approach may be of use for a clinical setting application, in which a quantitative measure may help the physician’s decision-making process in a polypharmacy setting to manage ADRs. 

Another strength is that the model may be readily adopted into a research and development (R&D) setting to analyze the effects of drug combinations. There are opportunities in such a setting for the quantification of functional processes, using either high-throughput means (such as the LINCS project that was used for this work) or using in vivo or in vitro models in experiments designed ad hoc for specific drugs and applications. Similarly, the quantification of ADRs could be measured in biological models or through the use of pharmacovigilance data. 

In this regard, it is important to have two limitations in mind. First, the tripartite network model reliability is heavily dependent on the reliability of the used data. This is especially important when using a large-scale, high-throughput data set, and adequate quality assurance and validation are needed. Secondly, if the input data for the model is of a different nature (for instance, ADR incidences from pharmacovigilance data and physiological measurements of an ADR in an in vivo model), it is not trivially possible to compare the insights obtained from the model. 

In conclusion, we consider that the model is capable of highlighting associations between the functional effects of a drug and the manifestation of ADRs. These highlighted drug–ADR associations may serve as leads to identifying mechanisms through which the drug may generate the adverse effects. Nevertheless, appropriate experiments must be performed to confirm the proposed associations. We propose that our method may be of use for pharmacological researchers for hypothesis generation and guiding experimental designs. 

## 4. Materials and Methods

### 4.1. Network Construction

#### Drug–GO Network:

We used the drug perturbation data analyzed in a previous study [9], downloaded from http://maayanlab.net/SEP-L1000/#download. Briefly, in that work, the researchers used principal angle enrichment analysis (PAEA) [15] to generate the perturbation signature of Gene Ontology terms for each drug analyzed. To identify the most significant GO terms for each drug, we used an implementation of ABC analysis [16] to select the subset of GO terms that were more significantly perturbed by each drug. We then constructed a bipartite network by representing drugs and GO terms as nodes and linking them if the GO term belonged to the significantly perturbed set of a given drug. This bipartite network was processed using the *Igraph* package [17] for R, as well as NetworkX [18] for Python for basic network properties.

### 4.2. Network Integration

The two bipartite networks were merged. GO terms that were not associated with at least one drug and one ADR were removed from the network, as they provided no information on possible mechanisms for the induction of ADRs by a given drug. The links in the network were given directions: drugs to GO terms and GO terms to ADRs.

### 4.3. Analysis of Drug Combinations from a Network Perspective

The main purpose of this work was to provide tools to analyze ADRs that may emerge in a drug combination setting. For this, we used the tripartite Drug–GO–ADR network and defined a series of features that may be extracted from this network for any combination of drugs reported in the network.

#### 4.3.1. Drug Combination Subgraph

Given a set of drugs, it is possible to extract from the tripartite graph a subgraph that contains the directed second-order neighborhoods of each drug in the combination. This subgraph represents the landscape of all possible functional perturbations and ADRs for that drug combination that are represented in the tripartite network model.

#### 4.3.2. Composite ADRs

Given a drug combination subgraph, it is possible to identify ADRs that may be generated by more than one drug. We refer to these as composite ADRs (cADRs). Composite ADRs may present a problem in a polypharmacy setting, as they may be generated by any of the drugs in the treatment regime; therefore, deciding which drugs (if any) should be suspended (or changed) to deal with the ADR is not possible, unlike with a noncomposite ADR, which may only be associated with a single drug (which may be substituted). There are two configuration modes that lead to the emergence of cADRs:

##### Mode 1

In mode 1, the same biological function (or functions) may be perturbed by more than one drug in the combination. The increased likelihood of functional perturbation leads to increased risk of the ADRs associated with said perturbation.

If the contribution to pathway perturbation of each drug is quantitatively known, and the contribution of each pathway to the ADR is also known, then it is possible to quantify the ADR “activity” for the drug combination as:

(2) $$\begin{array}{ll} Activity\left(ADR\right)= & Activity(ADR|\;Pathway)\times \;[Perturbation(Pathway|\;Drug\_1\;)\\ & +Perturbation(Pathway|Drug\_2\;)+\;\cdots \\ & +Perturbation(Pathway|\;Drug\_n\;)] \end{array}$$

##### Mode 2

In mode 2, each drug affects a different biological function (or functions), each of which is associated with the same ADR. Each perturbed biological function may independently increase the risk for the appearance of the ADR. Again, if the quantitative contributions to pathway perturbation by drugs and to ADR manifestation by pathway perturbation are known, it is possible to quantify a given ADR “activity” as:

(3) $$\begin{array}{ll} Activity\left(ADR\right)= & Activity(ADR_{1}|Pathway_{1})\;\times Perturbation\left(Pathway_{1}|Drug_{1}\right)\\ & +Activity\left(ADR|Pathway_{2}\right)\times Perturbation\left(Pathway_{2}|Drug_{2}\right)+\;\cdots \\ & +Activity\left(ADR|Pathway_{i}\right)\;\times \;Perturbation\left(Pathway_{i}|Drug_{n}\right) \end{array}$$

It should be noted that a given ADR may be associated with both mode 1 and mode 2 configurations, which may be described as a general model:

(4) $$Activity\left(ADR\right)=\;\underset{pathway}{\sum }Activity\left(ADR|Pathway_{i}\right)\;\underset{drug}{\sum }Perturbation\left(Pathway_{i}|Drug_{n}\right)$$

#### 4.3.3. Composite Risk Module

For a given set of composite ADRs, there will be a corresponding set of associated biological functions. Some composite ADRs will be linked through associated biological functions. We define a composite risk module (CRM) as the set of ADRs and biological functions that are connected if the drug nodes are removed. The emergence of any ADR in a given CRM module may be explained only by the perturbation of functions in the same CRM. Emergence of an ADR in a CRM module may also involve the emergence of other ADRs in the same module by the perturbation of the same associated functions. A drug combination with more CRMs may have a larger number of independent sets of related ADRs.

### 4.4. Example Selection

In order to illustrate the results that may be obtained from the model, we selected two examples of drug combinations to analyze. The first one is a combination of two serotonergic agents, fluoxetine and phenelzine, which may cause serotonin syndrome, a well-known drug interaction with a pharmacodynamic origin. For the second example, in which we wanted to showcase a three-drug combination, we decided to select three commonly prescribed drugs, particularly in geriatric populations: omeprazole, captopril, and metformin. For both examples, we selected an ADR (blurred vision and dry mouth, respectively) that presented both mode 1 and mode 2 of composite ADR configuration.

## Figures and Tables

**Table 1:** Topological parameters of the Drug–GO–ADR tripartite network.

Nodes and Edges	Counts
Drug Nodes	3454
GO Nodes	323
ADR Nodes	111
Drug to GO Edges	258,793
GO to ADR Edges	419

**Table 2:** Topological parameters of the fluoxetine–phenelzine drug combination network.

Parameter	Values
GO terms	107
ADRs	72
cADRs	43
GO nodes in mode 1 configurations	32
GO nodes in mode 2 configurations	47
CRMs	24
Size of largest CRM (ADR/GO)	53 (20/33)

## Supplementary Materials

Supplementary materials, a network file (GML format) containing the tripartite network model for drugs, GO terms, and ADRs as well as a zip file containing high-resolution heatmap image files for Figure 3, Figure 4, Figure 5 and Figure 6, can be found at http://www.mdpi.com/1422-0067/20/2/386/s1.

## Abbreviations

ADR	Adverse Drug Reaction
cADR	Composite Adverse Drug Reaction
CMap	Connectivity Map
LD	Linear dichroism
CRM	Composite Risk Module
GO	Gene Ontology
LINCS	Library of Integrated Network-Based Cellular Signatures

## References

[1] de Anda-Jauregui G., Guo K., McGregor B.A., Hur J. (2018). Exploration of the Anti-Inflammatory Drug Space Through Network Pharmacology: Applications for Drug Repurposing. Front. Physiol..

[2] Sultana J., Cutroneo P., Trifiro G. (2013). Clinical and economic burden of adverse drug reactions. J. Pharmacol. Pharm..

[3] Maher R.L., Hanlon J., Hajjar E.R. (2014). Clinical consequences of polypharmacy in elderly. Expert Opin. Drug Saf..

[4] Harris M.A., Clark J., Ireland A., Lomax J., Ashburner M., Foulger R., Eilbeck K., Lewis S., Marshall B., Mungall C. (2004). The Gene Ontology (GO) database and informatics resource. Nucleic Acids Res..

[5] Lamb J. (2007). The Connectivity Map: A new tool for biomedical research. Nat. Rev. Cancer.

[6] Keenan A.B., Jenkins S.L., Jagodnik K.M., Koplev S., He E., Torre D., Wang Z., Dohlman A.B., Silverstein M.C., Lachmann A. (2018). The Library of Integrated Network-Based Cellular Signatures NIH Program: System-Level Cataloging of Human Cells Response to Perturbations. Cell Syst..

[7] Subramanian A., Narayan R., Corsello S.M., Peck D.D., Natoli T.E., Lu X., Gould J., Davis J.F., Tubelli A.A., Asiedu J.K. (2017). A Next Generation Connectivity Map: L1000 Platform and the First 1,000,000 Profiles. Cell.

[8] Reddy A.S., Zhang S. (2013). Polypharmacology: Drug discovery for the future. Expert Rev. Clin. Pharmacol..

[9] Wang Z., Clark N.R., Ma’ayan A. (2016). Drug-induced adverse events prediction with the LINCS L1000 data. Bioinformatics.

[10] De Domenico M., Solé-Ribalta A., Cozzo E., Kivelä M., Moreno Y., Porter M.A., Gómez S., Arenas A. (2013). Mathematical Formulation of Multilayer Networks. Phys. Rev. X.

[11] Tatonetti N.P., Ye P.P., Daneshjou R., Altman R.B. (2012). Data-driven prediction of drug effects and interactions. Sci. Transl. Med..

[12] Zitnik M., Agrawal M., Leskovec J. (2018). Modeling polypharmacy side effects with graph convolutional networks. Bioinformatics.

[13] Bartlett D. (2017). Drug-Induced Serotonin Syndrome. Crit. Care Nurse.

[14] Teare J.P., Spedding C., Whitehead M.W., Greenfield S.M., Challacombe S.J., Thompson R.P. (1995). Omeprazole and dry mouth. Scand. J. Gastroenterol..

[15] van Dijk S.J., Molloy P., Varinli H., Morrison J., Muhlhausler B., Buckley M., Clark S., McMillen I., Noakes M., Samaras K. (2015). Epigenetics and human obesity. Int. J. Obes..

[16] Ultsch A., Lotsch J. (2015). Computed ABC Analysis for Rational Selection of Most Informative Variables in Multivariate Data. PLoS ONE.

[17] Csardi G., Nepusz T. (2006). The igraph software package for complex network research. InterJournal Complex Syst..

[18] Hagberg A., Swart P., Chult D.S. (2008). Exploring Network Structure, Dynamics, and Function Using NetworkX.

