# Supplementary material for: Network-Based Assessment of Adverse Drug Reaction Risk in Polypharmacy Using High-Throughput Screening Data
Source: Int J Mol Sci. 2019 Jan 17;20(2):386. doi: 10.3390/ijms20020386 (PMC6358820; doi:10.3390/ijms20020386)
Supplement: Supplementary file 1 [file ijms-20-00386-s001.zip › SupplementaryMaterials/SupplementaryFile02.zip!/SupplementaryFile02/SupplementaryFile02.zip!/Figure3B.pdf]

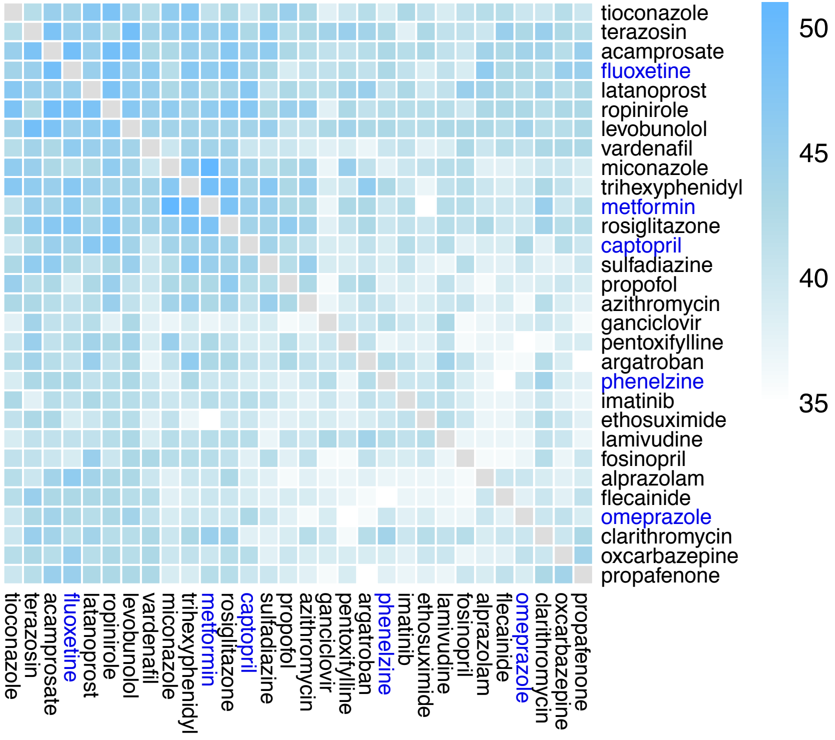

tioconazole
terazosin
acamprosate
fluoxetine
latanoprost
ropinirole
levobunolol
vardenafil
miconazole
trihexyphenidyl
metformin
rosiglitazone
captopril
sulfadiazine
propofol
azithromycin
ganciclovir
pentoxifylline
argatroban
phenelzine
imatinib
ethosuximide
lamivudine
fosinopril
alprazolam
flecainide
omeprazole
clarithromycin
oxcarbazepine
propafenone
50
45
40
35
