# Supplementary material for: Network-Based Assessment of Adverse Drug Reaction Risk in Polypharmacy Using High-Throughput Screening Data
Source: Int J Mol Sci. 2019 Jan 17;20(2):386. doi: 10.3390/ijms20020386 (PMC6358820; doi:10.3390/ijms20020386)
Supplement: Supplementary file 1 [file ijms-20-00386-s001.zip › SupplementaryMaterials/SupplementaryFile02.zip!/SupplementaryFile02/SupplementaryFile02.zip!/Figure4B.pdf]

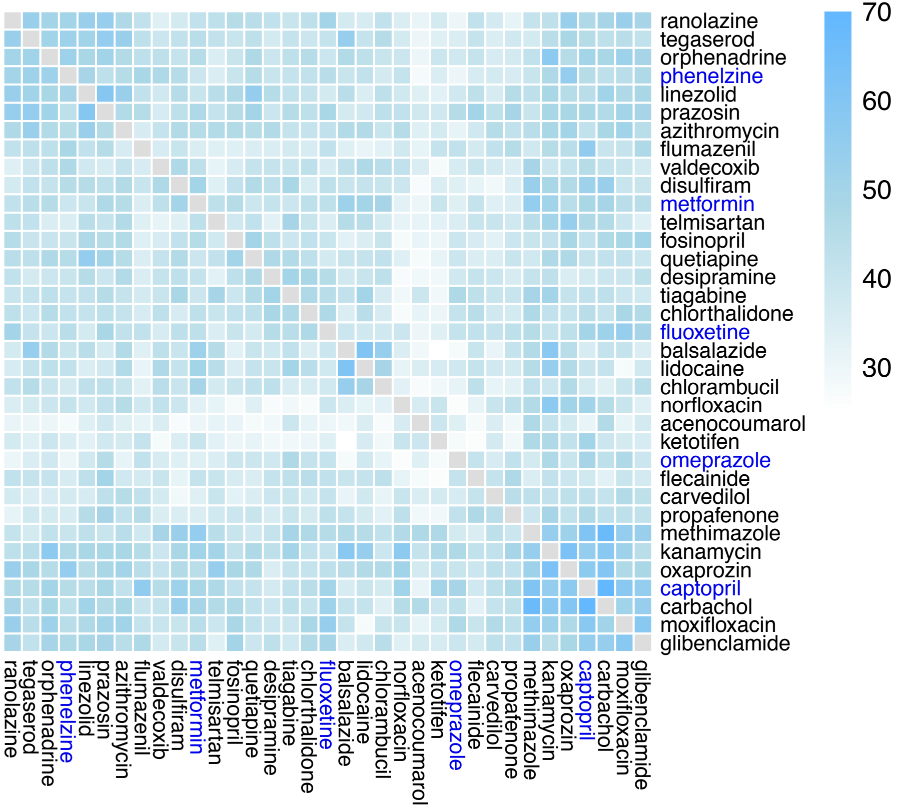
ranolazine
tegaserod
orphenadrine
phenelzine
linezolid
prazosin
azithromycin
flumazenil
valdecoxib
disulfiram
metformin
telmisartan
fosinopril
quetiapine
desipramine
tiagabine
chlorthalidone
fluoxetine
balsalazide
lidocaine
chlorambucil
norfloxacin
acenocoumarol
ketotifen
omeprazole
flecainide
carvedilol
propafenone
methimazole
kanamycin
oxaprozin
captopril
carbachol
moxifloxacin
glibenclamide
70
60
50
40
30
