# Supplementary material for: Network-Based Assessment of Adverse Drug Reaction Risk in Polypharmacy Using High-Throughput Screening Data
Source: Int J Mol Sci. 2019 Jan 17;20(2):386. doi: 10.3390/ijms20020386 (PMC6358820; doi:10.3390/ijms20020386)
Supplement: Supplementary file 1 [file ijms-20-00386-s001.zip › SupplementaryMaterials/SupplementaryFile02.zip!/SupplementaryFile02/SupplementaryFile02.zip!/Figure5A.pdf]

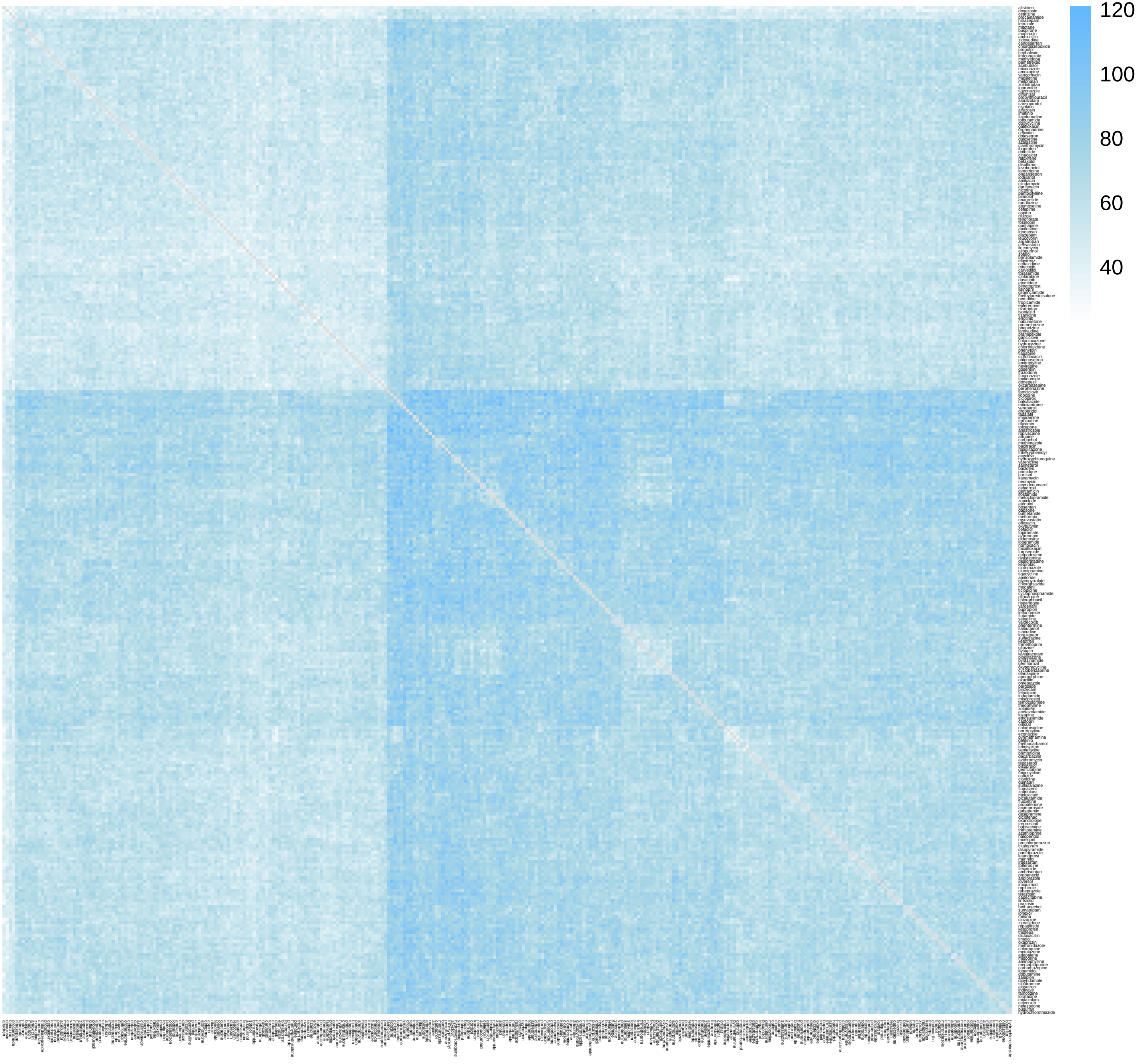
120
100
80
60
40
