# Supplementary material for: Network-Based Assessment of Adverse Drug Reaction Risk in Polypharmacy Using High-Throughput Screening Data
Source: Int J Mol Sci. 2019 Jan 17;20(2):386. doi: 10.3390/ijms20020386 (PMC6358820; doi:10.3390/ijms20020386)
Supplement: Supplementary file 1 [file ijms-20-00386-s001.zip › SupplementaryMaterials/SupplementaryFile02.zip!/SupplementaryFile02/SupplementaryFile02.zip!/Figure5B.pdf]

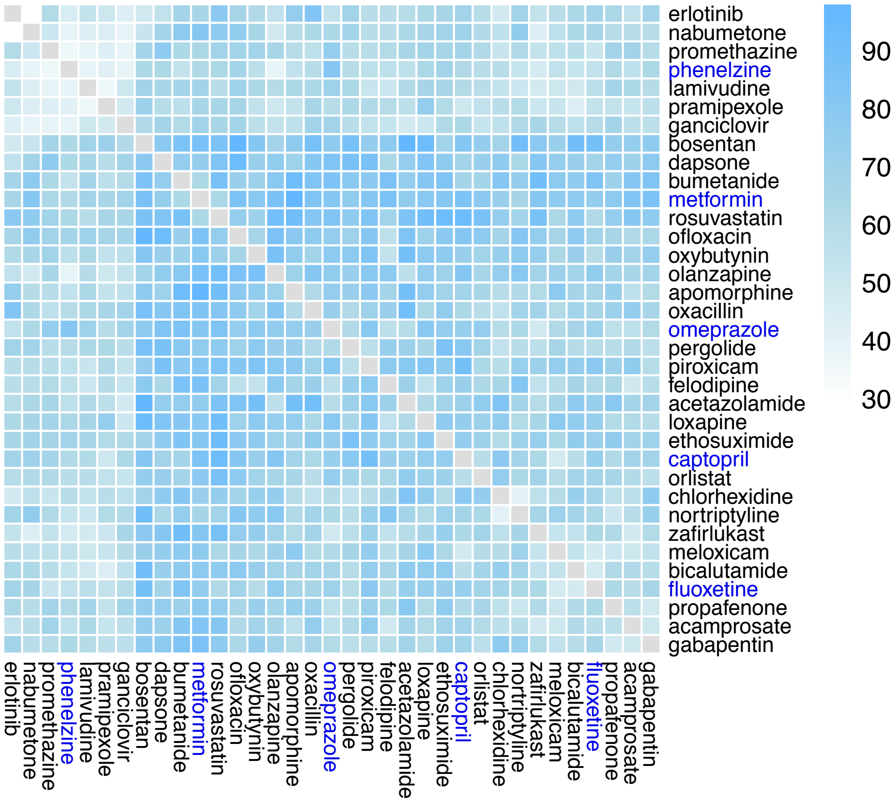
erlotinib
nabumetone
promethazine
phenelzine
lamivudine
pramipexole
ganciclovir
bosentan
dapsone
bumetanide
metformin
rosuvastatin
ofloxacin
oxybutynin
olanzapine
apomorphine
oxacillin
omeprazole
pergolide
piroxicam
felodipine
acetazolamide
loxapine
ethosuximide
captopril
orlistat
chlorhexidine
nortriptyline
zafirlukast
meloxicam
bicalutamide
fluoxetine
propafenone
acamprosate
gabapentin
90
80
70
60
50
40
30
