# Supplementary material for: Network-Based Assessment of Adverse Drug Reaction Risk in Polypharmacy Using High-Throughput Screening Data
Source: Int J Mol Sci. 2019 Jan 17;20(2):386. doi: 10.3390/ijms20020386 (PMC6358820; doi:10.3390/ijms20020386)
Supplement: Supplementary file 1 [file ijms-20-00386-s001.zip › SupplementaryMaterials/SupplementaryFile02.zip!/SupplementaryFile02/SupplementaryFile02.zip!/Figure6B.pdf]

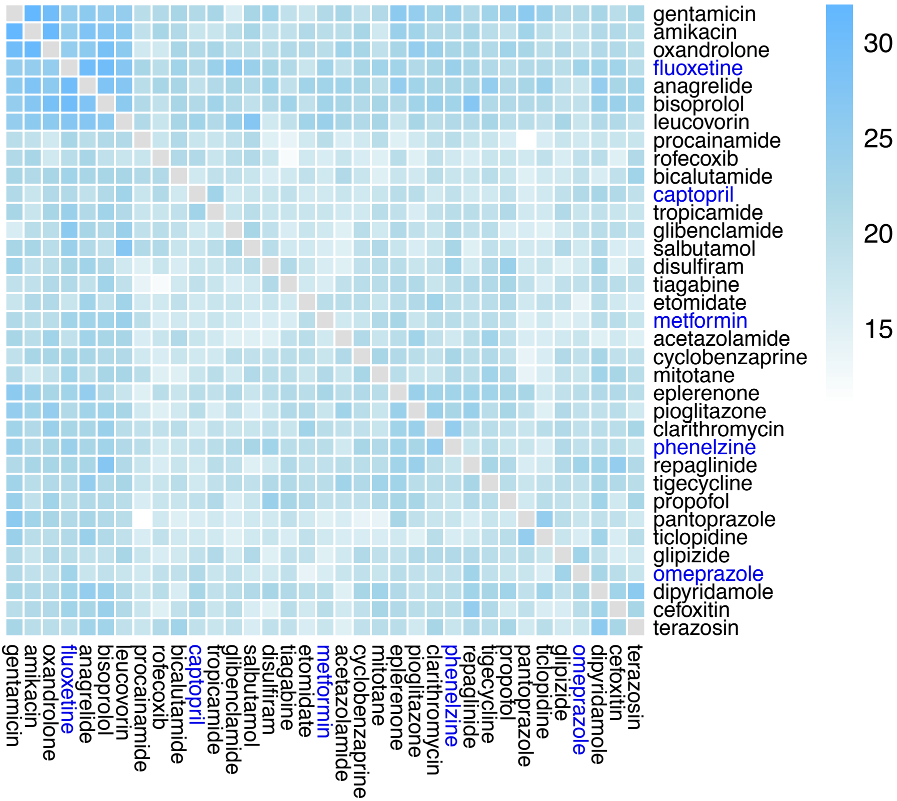
gentamicin
amikacin
oxandrolone
fluoxetine
anagrelide
bisoprolol
leucovorin
procainamide
rofecoxib
bicalutamide
captopril
tropicamide
glibenclamide
salbutamol
disulfiram
tiagabine
etomidate
metformin
acetazolamide
cyclobenzaprine
mitotane
eplerenone
pioglitazone
clarithromycin
phenelzine
repaglinide
tigecycline
propofol
pantoprazole
ticlopidine
glipizide
omeprazole
dipyridamole
cefoxitin
terazosin
30
25
20
15
